# The impact of chemical pollution and warming on male fertility: a narrative review by the Special Interest Group “Environment and Fertility” of the Italian Society of Fertility and Sterility and Reproductive Medicine (SIFES-MR)

**DOI:** 10.1007/s10815-025-03678-0

**Published:** 2025-10-09

**Authors:** Giuseppe Defeudis, Cristina de Angelis, Rossella Mazzilli, Federica Barbagallo, Claudia Leanza, Iva Sabovic, Rosita A. Condorelli, Rocco Rago, Daniele Gianfrilli, Rosario Pivonello, Andrea Di Nisio, Paola Anserini, Carlo Foresta, the Group of Special Interest for Environment of the Italian Society of Fertility, Sterility, Reproductive Medicine (SIFES-MR)

**Affiliations:** 1 Department of Theoretical and Applied Sciences, eCampus University, Novedrate CO, Italy; 2 Dipartimento di Medicina Clinica e Chirurgia, Sezione di Endocrinologia, Diabetologia, Andrologia e Nutrizione, Unità di Andrologia e Medicina della Riproduzione, Sessualità e Affermazione di Genere, Università Federico II di Napoli, Naples, Italy; 3 Department of Clinical and Molecular Medicine, Sapienza University of Rome, Rome, Italy; 4 IVIRMA Global Research Alliance, Genera, Clinica Valle Giulia, Rome, Italy; 5 Department of Clinical and Experimental Medicine, University of Catania, Catania, Italy; 6 Unit of Andrology and Medicine of Human Reproduction, Department of Medicine – DIMED, University of Padova, Via Giustiniani, 2, Padua, 35128 Italy; 7 Physiopathology of Reproduction and Andrology Unit, Sandro Pertini Hospital, Rome, Italy; 8 Department of Experimental Medicine, University Sapienza of Rome, Rome, Italy; 9 Staff of UNESCO Chair for Health Education and Sustainable Development, Federico II University, Naples, Italy; 10 Department of Psychology and Health Sciences, Pegaso University, Naples, Italy; 11 SSD Fisiopatologia Della Riproduzione Umana (FRU) IRCCS Ospedale Policlinico San Martino Di Genova, Genoa, Italy; 12 Department of Experimental Medicine, Section of Medical Pathophysiology, Endocrinology and Food Sciences, Sapienza University of Rome, Rome, Italy

**Keywords:** Endocrine disruptors (EDC), Air pollution, Global warming, Sperm, Male fertility

## Abstract

Environmental changes are a growing global concern, and their impact on reproductive health remains incompletely understood. In this narrative review, conducted on behalf of the Italian Society of Fertility and Sterility and Reproductive Medicine (SIFES-MR), we examined the impact of the environment on male fertility, considering endocrine-disrupting chemicals (EDCs), air pollution, and global warming, with the aim of identifying strategies to improve reproductive outcomes. Scientific literature demonstrates that all these aspects may contribute to a decline in reproductive health, impairing sperm count, motility, and morphology as well as reducing testicular hormonal function. Future research should focus on the role of environmental factors in male hypogonadism, impaired spermatogenesis, genital abnormalities, and transgenerational effects.

## Introduction

Radical societal changes of the twenty-first century are expected to significantly affect global fertility trends. It is estimated that in 2050, the fertility rate of almost all nations will fall below 2.1 children per woman; in Italy, the average will be even lower, standing at 1.18, and down to 1.09 in 2100. Several lines of evidence support the hypothesis that declines in fertility rates might be related to environmental factors; in particular, exposure to chemicals in fetal life may also be dangerous [1].

The exposure to several chemical and physical pollutants, including heavy metals, pesticides, phthalates, and other endocrine-disrupting chemicals (EDCs) found in air, water, and soil, has been associated with impaired gametogenesis, both in males and females.

In the absence of risk-reduction actions, exposure to EDCs contributes at least 20% to the incidence of reproductive diseases, and for the general population, food is the main vehicle for exposure to these chemicals.

Populations residing in heavily industrialized or agricultural regions are especially vulnerable due to higher levels of chemical exposure. Therefore, the European Union considers the identification of EDCs on the European market a priority (Article 57f of the REACH Regulation) and has promoted the monitoring of food contamination and environmental pollution. In addition, the World Health Organization (WHO) recognizes global warming as a major threat to human health, including reproductive health [2].

This review conducted by the “Environment and Fertility” Special Interest Group (GIS) of the Italian Society of Fertility, Sterility and Reproductive Medicine (SIFES-MR)—comprising experts in andrology, reproductive medicine, biology, embryology, and assisted reproductive technologies—aims to evaluate the impact of environmental exposures, including EDCs, air pollution, and global warming, on male fertility in order to organize preventive and therapeutic programs. For this purpose, we completed a retrospective analysis of human studies published in the last 10 years.

A summary at a glance of the human studies considered in the current review is provided in and Table 1 for chemical pollutant, Table 2 for air pollutant, and Table 3 for global warming.

**Table 1:** Summary at a glance of clinical studies on the effects of chemical pollutants on male reproductive function

Ref	Study design	Chemical pollutant	N° of subjects Type of exposure	Type of patients N° of subjects/group	Age (years)	Biological matrix for dosage	Main findings
Ramsay JM et al. (2023)	Ecological retrospective cross-sectional study	Airborne EDCs from industrial sources estimated by Risk-Screening Environmental Indicators Geographic Microdata (RSEI-GM) model	21.563 Environmental	Male partners of infertile couples	M 31 ± 6.4	-	Decreased sperm total motility across heavy metals and phthalates quartiles of exposure Decreased semen volume across phthalates quartiles of exposure Increased odds for azoospermia across phthalates quartiles of exposure
Perrone P et al. (2022)	Prospective cross-sectional study	Volatile organic compounds	156 Environmental	Males from the general population **Group 1:** 80–low pollution area **Group 2:** 76–high pollution area	R 18–30	Sem	**Group 2:** Decreased sperm concentration and total motility. Increased non-progressive and immotile spermatozoa, abnormal sperm morphology Decreased protamine/histone ratio and DNA-binding ability
Huang X et al. (2019)	Ecological prospective cross-sectional study	PM2.5 and its constituents	1.081 Environmental	Male partners of infertile couples	R 22.2–58.6 M 34.7 ± 5.5	-	+ Cd, + Pb associated with decreased sperm concentration
Buck Louis GM et al. (2016)	Prospective cross-sectional study	Heavy metals, PCBs, PFAS, BPA, phthalates	401 couples Environmental	Male partners of couples discontinuing contraception for pregnancy planning enrolled from the Longitudinal Investigation of Fertility and the Environment (LIFE) study cohort **Group 1:** 347–couples achieving pregnancy **Group 2:** 54–couples not achieving pregnancy	≥ 18 **Group 1:** M 31.6 ± 4.6 **Group 2:** M 32.4 ± 5.3	B (Heavy metals) S (PCB, PFAS) Ur (BPA, phthalates)	+ Male partners’ B Pb, Ur MBzP, Ur MMP associated with decreased couple fecundity, even after adjusting for female partners’ concentrations
Sukhn C et al. (2018)	Prospective cross-sectional study	Heavy metals	116 Environmental	Male partners of infertile couples **Group 1:** 61–at least 1 seminal parameter below-reference **Group 2:** 55–normozoospermia	R 18–55 **Group 1:** M 37.4 ± 0.8 **Group 2:** M 37.6 ± 0.8	B Sem	**Group 1:** + Sem Cd, + Sem Ba + B Cd, + B Ba, + Sem Pb, + Sem Cd, + Sem Ba, + Sem U associated with below-reference sperm viability + Sem U associated with below-reference progressive sperm motility and normal morphology
Olszak-Wasik K et al. (2022)	Prospective cross-sectional study	Heavy metals (Pb, Cd)	188 Environmental	Male partners of infertile couples **Group 1:** 91–agricultural area (32 class 1, 54 class 2)* **Group 2:** 97–industrial area (59 class 1, 32 class 2)*	**Group 1:** M 33.3 ± 3.9 **Group 2:** M 89 34.9 ± 4.9	Sem	+ Sem Cd associated with poor semen quality, independently from the agricultural or industrial residential address
Hassan J et al. (2024)	Prospective cross-sectional case–control study	Cd	66 Environmental	**Group 1:** 33–fertile **Group 2:** 33–idiopathic infertility	R 25—45	B Sem	**Group 2:** + B Cd, + Sem Cd B Cd ↓ blood GSH and ↑ serum MDA
Pant N et al. (2015)	Prospective cross-sectional case–control study	Heavy metals (Pb, Cd)	119 Environmental	**Group 1:** 46–fertile **Group 2:** 73–infertile	R 21–40 **Group 1:** M 31.82 ± 6.56 **Group 2:** M 32.90 ± 5.94	Sem	**Group 2:** + Sem Pb, + B Cd, + Sem Cd + Sem Pb, + Sem Cd associated with below-reference sperm total count and motility
Chinyere Nsonwu-Anyanwu A et al. (2019)	Prospective cross-sectional study	Heavy metals (Pb, Cd)	130 Environmental	Male partners of infertile couples **Group 1:** 30–azoospermia **Group 2:** 50–oligozoospermia **Group 3:** 50–normozoospermia	R 20–60 **Group 1:** M 32.85 ± 3.22 **Group 2:** M 33.57 ± 3.77 **Group 3:** M 34.26 ± 4.04	S Sem	**Group 1:** + B Pd, + B Cd vs group 3 **Group 2:** + B Pd, + B Cd vs group 3
Famurewa AC et al. (2017)	Prospective cross-sectional study	Heavy metals (Pb, Cd)	73 Environmental	Male partners of infertile couples **Group 1:** 15–azoospermia **Group 2:** 22–oligozoospermia **Group 3:** 36–normozoospermia	R 20–45 M 37.1 ± 7.0 **Group 1:** M 39.54 ± 7.30 **Group 2:** M 37.32 ± 6.90 **Group 3:** M 36.08 ± 6.96	B Sem	**Group 1:** + B Pd, + B Cd, + Sem Cd vs group 3 **Group 2:** + B Pd, + B Cd, + Sem Cd vs group 3 B Cd and Sem Cd ↓ sperm concentration, motility, morphology B Pb ↓ sperm concentration
Mitra S et al. (2020)	Prospective cross-sectional study	Heavy metals (Pb, Cd)	400 Occupational	**Group 1:** 200–tea garden workers from infertile couples **Group 2:** 200–non tea garden workers, normozoospermia	R 21–40	Sem	**Group 1:** + Sem Pd, + Sem Cd Sem Pb and Sem Cd ↓ sperm progressive motility, morphology Sem Pb and Sem Cd ↑ seminal MDA, SOD, sperm DNA damage, p53 and ↓ seminal GSH, TACs, p-Akt, NFκB, Bcl2
Wang X et al. (2016)	Prospective cross-sectional case–control study	As	162 Environmental	**Group 1:** 101–infertile **Group 2:** 61–fertile	R 19–43 **Group 1:** M 28.7 ± 3.9 **Group 2:** M 28.5 ± 5.3	Ur	**Group 1:** + Ur As + Ur As associated with unexplained infertility
Minguez-Alarcon L et al. (2018)	Prospective cross-sectional study	Hg	129 Environmental	Male partners of infertile couples	R 18–56 m 36	H	+ H Hg associated with increased sperm concentration, total count, progressive motility Associations were attenuated by controlling for fish consumption and were no longer significant
Benson TE et al. (2021)	Prospective cross-sectional study	BPA, BPF, BPS	556 Environmental	Males from the general population enrolled from the Fetal Programming of Semen Quality (FEPOS) cohort, a sub-study within The Danish National Birth Cohort (DNBC)	R 18–20	Ur	Ur BPA, Ur BPF, Ur BPS not associated with seminal parameters
Ji H et al. (2018)	Prospective cross-sectional study	BPA	500 Environmental	Males from the general population who had fathered at least one child **Group 1:** 132–undetected Ur BPA **Group 2:** 368–detected Ur BPA	R 18–55	Ur	**Group 2:** Decreased sperm concentration, ALH, MAD and increased LIN, STR, WOB
Pollard SH et al. (2019)	Prospective cross-sectional study	BPA	161 Environmental	Male partners of couples enrolled in Home Observation of Periconceptional Exposures (HOPE) study	M 28.5 ± 3.9	Ur	+ Ur BPA associated with abnormal sperm morphology (tail defects)
Mantzouki C et al. (2019)	Prospective cross-sectional case–control study	BPA	80 Environmental	**Group 1:** 55–infertile **Group 2:** 25–fertile	**Group 1:** Subgroup INOA M 34.6 ± 1.6 Subgroup grade II or III varicocele and OAT M 37.4 ± 1.3 Subgroup cryptorchidism M 33.7 ± 1.2 **Group 2:** M 30.7 ± 1.7	S	S BPA detected at comparable levels in group 1 and 2; high S BPA (> 3 ng/ml) specifically detected only in group 1
Caporossi L et al. (2022)	Retrospective cross-sectional case–control study	BPA, phthalates	366 Environmental	**Group 1:** 155–infertile **Group 2:** 211–fertile	**Group 1:** R 30–67 m 40.4 **Group 2:** R 22–58 m 36.1	Ur	Ur BPA, Ur MEP detected at comparable levels in group 1 and 2 **Group 1:** + Ur MBP, + Ur MBzP, + Ur MOP, + Ur DEHP Ur BPA, Ur MBP, Ur MEP, Ur MBzP, Ur MOP, Ur DEHP not associated with seminal parameters
Omran GA et al. (2018)	Prospective cross-sectional case–control study	BPA	100 Environmental	**Group 1:** 50–infertile **Group 2:** 50–fertile	R 20–54	Ur	Ur BPA detected at comparable levels in group 1 and 2 **Group 1:** Ur BPA ↓ sperm concentration, motility, morphology Ur BPA ↑ DNA damage and MDA **Group 2:** Ur BPA ↓ sperm concentration, motility, morphology, antioxidants Ur BPA ↑ DNA damage and MDA
Chen PP et al. (2022)	Prospective cross-sectional study	BPA, BPF, BPS	984 Environmental	Male partners of couples attending Reproductive Center for semen analysis	M 32.0 ± 5.4	Ur	+ Ur BPA associated with below-reference sperm concentration, total count and total and progressive motility + Ur BPS associated with below-reference sperm total and progressive motility
Radwan M et al. (2018)	Prospective cross-sectional study	BPA	315 Environmental	Males attending Reproductive Center for semen analysis	M 32.14 ± 4.23	Ur	+ Ur BPA associated with increased total sperm sex chromosome disomy and spermatozoa immaturity
Caporossi L et al. (2020)	Prospective cross-sectional study	BPA, phthalates	105 Environmental	Male partners of infertile couples	R 29–67 m 40.5	Ur	Ur BPA not associated with seminal parameters Ur MBP, Ur MOP, Ur MINP ↓ semen volume Ur MEP ↓sperm concentration
Den Hond E et al. (2015)	Prospective cross-sectional case–control study	PCB, dioxin, PFAS, BPA, phthalates	120 Environmental	**Group 1:** 80–fertile **Group 2:** 40–infertile	**Group 1:** M 34.1 **Group 2:** M 31.6	S (PCB, dioxin, PFAS) Ur (BPA, phthalates)	Ur BPA, Ur MEHP, Ur MEP, Ur MBP, Ur MIBP, Ur MBzP not associated with seminal parameters
Ghayda RA et al. (2019)	Prospective cross-sectional study	BPS	158 Environmental	Male partners of infertile couples enrolled in Environment and Reproductive Health (EARTH) study	R 18–56 m 35.6	Ur	Decreased semen volume and sperm concentration in men with Ur BPS > LOD vs Ur BPS < LOD When stratifying for BMI, only in overweight and obese men: decreased sperm concentration, total count and motility in men with Ur BPS > LOD vs Ur BPS < LOD
Vitku J et al. (2016)	Prospective cross-sectional study	PCB, BPA	191 Environmental	Male partners of infertile couples	M 35.8 ± 5.6	P (PCB, BPA) Sem (BPA)	P BPA ↑ sperm non-progressive motility Sem BPA ↓ sperm concentration, total count, and morphology
Jeseta M et al. (2024)	Prospective cross-sectional study	BPA, BPF, BPS	349 Environmental	Male partners of infertile couples	m 32	Sem	Sem BPA ↓sperm progressive motility and morphology Sem BPS ↓ semen volume and sperm total count
Buck Louis GM et al. (2014)	Prospective cross-sectional study	BPA, phthalates	501 couples Environmental	Male partners of couples discontinuing contraception for pregnancy planning enrolled from the Longitudinal Investigation of Fertility and the Environment (LIFE) study cohort	≥ 18 M 31.8 ± 4.9	Ur (BPA, phthalates)	Male partners' Ur BPA not associated with couple fecundity + Male partners' Ur MBzP, Ur MMP associated with decreased couple fecundity, even after adjusting for female partners’ concentrations
Buck Louis GM et al. (2018)	Prospective cross-sectional study	BPA, BPS, BPF, phthalates	339 couples Environmental	Male partners of couples discontinuing contraception for pregnancy planning enrolled from the Longitudinal Investigation of Fertility and the Environment (LIFE) study cohort **Group 1:** 246–couples achieving pregnancy **Group 2:** 93–couples not achieving pregnancy	≥ 18 **Group 1:** M 31.4 ± 0.3 **Group 2:** M 32.2 ± 0.6	Sem	Male partners’ Sem BPA, Sem BPS, Sem BPF not associated with couple fecundity Male partners’ Sem MECPP, Sem MCMHP, Sem MEOHP, Sem MEHHP, Sem MCPP, Sem MMP, Sem MEP, Sem MBP, Sem MIBP, Sem MHxP, Sem MCHP, Sem MOP, Sem MINP, Sem MBzP, Sem MIDP, Sem DMP, Sem DEP, Sem DIBP, Sem DBP, Sem BBP, Sem DNHP, Sem DCHP, Sem DEHP, Sem DOP not associated with couple fecundity
Yeum D et al. (2019)	Prospective cross-sectional study	BPA	164 couples Environmental	Male partners of couples enrolled in Home Observation of Periconceptional Exposures (HOPE) study	R 18–40 M 28.6 ± 3.8	Ur	Male partners’ Ur BPA not associated with couple fecundity
Goldstone AE et al. (2015)	Prospective cross-sectional study	BPA	418 couples Environmental	Male partners of couples discontinuing contraception for pregnancy planning enrolled from the Longitudinal Investigation of Fertility and the Environment (LIFE) study cohort	≥ 18 M 31.7 ± 4.9	Ur	Male partners’ Ur BPA not associated with seminal parameters with the exception of ↓ spermatozoa DNA fragmentation
Mínguez-Alarcón L et al. (2018)	Prospective longitudinal study	BPA, phthalates	936 Environmental	Male partners of infertile couples	R 18–56 m 35.7	Ur	Downward trends in sperm concentration and morphology over time (between year 2000 and 2017) were attenuated after adjusting for Ur phthalates; in particular, Ur MECPP) accounted for most of the contribution towards decreasing sperm concentration, and Ur MIBP) and Ur MCPP towards decreasing sperm morphology
Axelsson J et al. (2015)	Prospective cross-sectional study	Phthalates	314 Environmental	Males from the general population	R 17–20 M 18.4 ± 0.36	S Ur	+ Ur MECPP, + Ur MEHHP, + Ur MEOHP associated with decreased sperm progressive motility + Ur MEHP associated with increased spermatozoa immaturity
Chang WH et al. (2017)	Prospective cross-sectional study	Phthalates	290 Environmental	**Group 1:** 253–male partners of infertile couples (124–normozoospermia, 129–seminal parameters below-reference) **Group 2:** 37–male partners of fertile couples	**Group 1:** Normozoospermia M 33.3 ± 0.34 Below-reference M 35.4 ± 0.33 **Group 2:** M 32.7 ± 0.60	Sem Ur	+ Sem MEHP, + Sem MEHHP associated with decreased sperm concentration + Sem MEP, + Sem MEHHP, + Sem MECPP associated with decreased sperm motility + Sem MEP associated with decreased sperm morphology + Ur MBzP, + Ur MEHP associated with decreased sperm concentration and motility
Pan Y et al. (2015)	Prospective cross-sectional study	Phthalates	1.066 Environmental	Male partners of infertile couples including both couples with male factor and couples with female factor	m 29.1	Ur	+ Ur MBzP, associated with decreased sperm concentration and total count + Ur MBP, + Ur MIBP associated with decreased sperm morphology and increased spermatozoa DNA fragmentation + Ur MBP, + Ur MIBP, + Ur MEHP associated with decreased acrosin activity
Bloom MS et al. (2015)	Prospective cross-sectional study	Phthalates	473 couples Environmental	Male partners of couples discontinuing contraception for pregnancy planning enrolled from the Longitudinal Investigation of Fertility and the Environment (LIFE) study cohort	R 19–51 M 31.8 ± 4.9	Ur	+ Male partners’ Ur MCMHP, Ur MEHHP, Ur MBzP, Ur MNP associated with decreased sperm concentration and total count + Male partners' Ur MCMHP, Ur MEOHP, Ur MECPP, Ur MCPP associated with decreased sperm motility + Male partners' Ur MCMHP, Ur MNP associated with decreased sperm morphology
Cosci I et al. (2022)	Prospective cross-sectional case-control study	Phthalates	100 Environmental	**Group 1:** 78–fertile **Group 2:** 22–infertile	**Group 1:** M 33.4 ± 9.7 **Group 2:** M 39.2 ± 8.2	Sem	**Group 2:** + Sem DOP Sem DMP, Sem DEP, Sem DBP, Sem BBP, Sem DEHP, Sem DOP not associated with seminal parameters
Wang SY et al. (2015)	Prospective cross-sectional case-control study	Phthalates	201 Environmental	**Group 1:** 107–infertile **Group 2:** 94–fertile	R 23–45 M 25.51 ± 2.57	Sem	**Group 1:** + Sem DOP, + Sem DEP, + Sem BBP, + Sem DBP, + Sem DEHP
Rana R et al. (2020)	Prospective cross-sectional case-control study	Phthalates	302 Environmental	**Group 1:** 227–idiopathic infertility **Group 2:** 75–fertile	**Group 1:** R 18–45 M 30.38 ± 5.46 **Group 2:** R 18–45 M 31.57 ± 5.14	S Ur	**Group 1:** + S DEHP, + S DBP, + S DIBP, + S BEHIP, + S BPBG, + S DPP, + S DIOP, + S DIHP, + S DMP, + S DINP, + S BIOP, + S DMOP, + S DCHP + Ur MEHP, + Ur MBP None of the detected phthalates was associated with seminal parameters
Wang YX et al. (2015)	Prospective cross-sectional study	Phthalates	1.040 Environmental	Male partners of infertile couples including both couples with male factor and couples with female factor	M 32.09 ± 5.36	Ur	+ Ur MBP associated with below-reference sperm concentration and total count + Ur MEHP associated with abnormal sperm morphology
Wang YX et al. (2016)	Prospective cross-sectional study	Phthalates	687 Environmental	Male partners of infertile couples	M 32 ± 5.5	Sem	+ Sem MBP, + Sem MEHP, + Sem MEHHP, + Sem MEOHP associated with decreased semen volume + Sem MBzP, + Sem MEHP associated with decreased VCL, VSL + Sem MBzP associated with abnormal sperm morphology
Thurston SW et al. (2016)	Prospective cross-sectional study	Phthalates	420 Environmental	Male partners of pregnant females enrolled from the Study for Future Families (SFF)	R 32–53 M 32 ± 6	Ur	Ur MBzP ↓ sperm motility Ur DEHP, Ur DBP, Ur DEP, Ur DOP not associated with seminal parameters
Yin A et al. (2024)	Prospective cross-sectional study	Phthalates	251 couples Environmental	Male partners of couples discontinuing contraception for pregnancy planning enrolled from the Fudan Preconceptional Offspring Trajectory Study (PLOTS) cohort **Group 1:** 107–couples not achieving pregnancy **Group 2:** 144–couples achieving pregnancy	-	Ur	+ Male partners’ Ur total phthalates, Ur MEHP, Ur MCMHP associated with decreased couple fecundity
Specht IO et al. (2015)	Prospective cross-sectional study	Phthalates	401 Environmental	Male partners of pregnant females enrolled from the INUENDO Study cohort	≥ 18	S	+ Male partners’ S MEHP associated with either decreased or increased couple fecundity according to country
Den Hond E et al. (2015)	Case–control	PCBs	163 Environmental exposure	Subfertile men	< 50	Sem Ur	PCB exposure associated with altered fertility outcomes
Petersen MS et al. (2015)	Cross-sectional	PCBs	266 High environmental exposure	Fertile men	R 24.5–45.1 M 34.8	S Sem	+ PCB linked to ↑ SHBG and FSH, no effect on semen quality
Tøttenborg SS et al. (2022)	Cohort (prenatal)	PCBs	Maternal prenatal exposure	Offspring of exposed mothers	> 18 (adult follow up)	Maternal S (prenatal)	+ maternal PCB exposure → ↓ sperm count, ↑ FSH, ↑ risk of small testes, ↓ sperm DNA fragmentation index
Cai JL et al. (2015)	Observational	PCBs	36 Environmental exposure	Male patients	32.6 +/− 5.8	Sem	PCB exposure associated with ↓ prosaposin levels (marker of sperm maturation)
Moltó J et al. (2016)	Observational	PCBs	21 Environmental exposure	Male patients recruited for assisted reproduction	R 30–55	S	Higher PCB levels correlated with ↓ sperm quality (motility, morphology)
Desalegn AA et al. (2021)	Case-cohort	PCBs	Perinatal exposure of 641 male infants	Male offspring	Childhood 33 days after delivery Adulthood	Maternal serum and breastmilk/cord blood	Prenatal PCB exposure associated with ↑ risk of cryptorchidism and later reproductive disorders
La Rocca C et al. (2015)	Cross-sectional	PFAS (PFOA, PFOS)	Environmental 70	Men from different Italian regions	R 27–40	S Sem	+ PFAS exposure in metropolitan men → altered expression of nuclear receptors (androgen receptor)
Di Nisio A et al. (2019)	Observational	PFAS (PFOA)	Environmental 383	Male patients 171 control 212 exposure	18.5–22 (control) R 18–24 (exposed)	S Sem	PFOA interfered with androgen receptor → ↓ sperm count/quality, ↑ LH
Alamo A et al. (2024)	In vitro	PFAS (PFOA)	50 Laboratory exposure	healthy subjects	M 34.6 ± 3.2	Sem	+ PFOA reduced motility, ↑ lipid peroxidation, impaired chromatin
Šabović I et al. (2020)	Observational	PFAS	10 Environmental exposure	Male patients	R ≥ 20 and ≤ 35	Sem	PFAS exposure → reduced motility, impaired chromatin compactness, oxidative stress
Forthun IH et al. (2023)	Cohort	PFAS	Environmental exposure 449	Norwegian boys/youth	R 6–16	S	+ PFAS levels → altered pubertal timing, possible long-term reproductive impact
Forthun IH et al. (2024)	Cohort	PFAS	Environmental exposure 300	Norwegian boys	R 9–16	S	Higher PFAS exposure → delayed puberty onset
Paul R et al. (2017)	Case–control	Dioxin-like PCBs	Environmental exposure	Men attending fertility clinics	Adult	S	High dioxin-like PCB levels → delayed post-testicular sperm maturation
Chiu YH et al. (2015)	Prospective Cross-sectional study	Pesticides from fruit and vegetable intake estimated by data from the United States Department of Agriculture (USDA) Pesticide Data Program (PDP)	310 Environmental	Male partners of infertile couples enrolled in Environment and Reproductive Health (EARTH) study	R 26–51 m 36	-	Decreased sperm total count, motility, and normal morphology across quartiles of intake of high pesticide residue fruits and vegetables
Ghafouri-Khosrowshahi A et al. (2019)	Prospective Cross-sectional study	OP estimated by serum butyrylcholinesterase	60 Occupational	**Group 1:** 30–rural farmers **Group 2:** 30–urban men	R 18–30	-	**Group 1:** Decreased sperm concentration and total and progressive motility Decreased seminal TACs
Manikanda I et al. (2021)	Prospective Cross-sectional case–control study	OP estimated by serum pseudocholinesterase and acetylcholinesterase and urinary OP metabolites dialkyl phosphate	100 Environmental	**Group 1:** 50–male partners of infertile couples with below-reference seminal parameters **Group 2:** 50–normozoospermia	**Group 1:** M 34.94 ± 5.23 **Group 2:** M 34.20 ± 6.61	-	**Group 1:** Higher prevalence of OP exposure Urinary dialkyl phosphate ↓ sperm concentration, motility and normal morphology
Dziewirska E et al. (2019)	Prospective Cross-sectional study	OP estimated by urinary non persistent insecticide metabolites	315 Environmental	Male partners of infertile couples	< 45 M 32.14 ± 4.2	-	Urinary OP metabolites ↓ sperm motility, normal morphology and spermatozoa DNA fragmentation
Lin B et al. (2021)	Prospective cross-sectional study	p,p’-DDE	76 Environmental	Males from the general population	R 20–50 M 37.5 ± 8.6	S Sem	S p,p′-DDE ↓ sperm motility, ALH, BCF
Ghayda RA et al. (2020)	Prospective longitudinal study	OC	152 Environmental	Boys enrolled from the Russian Children’s Study (RCS)	R 8–9 at enrollment m 18.2 at semen analysis	S (at enrollment)	+ S HCB, + S βHCH associated with decreased semen volume + S p,p′-DDE associated with decreased sperm progressive motility
Mumford SL et al. (2015)	Prospective cross-sectional study	OC	468 couples Environmental	Male partners of couples discontinuing contraception for pregnancy planning enrolled from the Longitudinal Investigation of Fertility and the Environment (LIFE) study cohort	M 31.8 ± 4.8	S	+ Male partners’ S β-HCH associated with increased sperm motility, ALH, VAP, LIN, STR, VCL, VSL and percent cytoplasmic droplet, and decreased spermatozoa DNA stainability + Male partners' S p,p′-DDT, S o,p′-DDT, S p,p′-DDE associated with increased sperm motility + Male partners’ S o,p′-DDT associated with increased normal sperm morphology

*EDCs* endocrine-disrupting chemicals, *PM2.5* ambient fine particulate matter, *PCBs* polychlorinated biphenyls, *PFAS* perfluoroalkyl substances, *BPA* bisphenol A, *BPF* bisphenol F, *BPS* bisphenol S, *Pb* lead, *Cd* cadmium, *Ba* barium, *U* uranium, *As* arsenic, *Hg* mercury, *MBzP* monobenzyl phthalate, *MMP* monomethyl phthalate, *MEP* monoethyl phthalate, *MBP* monobutyl phthalate, *MOP* mono-octyl phthalate, *DEHP* di(2-ethylhexyl) phthalate, *MINP* mono-isononyl phthalate, *MECPP* mono(2-ethyl-5-carboxypentyl) phthalate, *MCMHP* mono[2-(carboxymethyl)hexyl] phthalate, *MEOHP* mono(2-ethyl-5-oxohexyl) phthalate, *MEHHP* mono(2-ethyl-5-hydroxyhexyl) phthalate, *MCPP* mono(3-carboxypropyl) phthalate, *MIBP* monoisobutyl phthalate, *MHxP* mono-hexyl phthalate, *MCHP* mono-cyclohexyl phthalate, *MIDP* mono-(8-methyl-1-nonyl) phthalate, *DMP* dimethyl phthalate, *DEP* diethyl phthalate, *DIBP* diisobutyl phthalate, *DBP* dibutyl phthalate, *BBP* benzyl butyl phthalate, *DNHP* di-n-hexyl phthalate, *DCHP* dicyclohexyl phthalate, *DOP* di-n-octyl phthalate, *MEHP* mono(2-ethylhexyl) phthalate, *MNP* monononyl phthalate, *BEHIP* bis(2-ethylhexyl) isophthalate, *BPBG* butyl phthalyl butyl glycollate, *DPP* dipentyl phthalate, *DIOP* diisooctyl phthalate, *DIHP* diisoheptyl phthalate, *DINP* diisononyl phthalate, *BIOP* butyl isooctyl phthalate, *DMOP* dimethoxy octyl phthalate, *R* range, *M* mean, *m* median, *BMI* body mass index, *INOA* idiopathic non-obstructive azoospermia, *OAT* oligo-astheno-teratozoospermia, *B* blood, *P* plasma, *S* serum, *Sem* semen, *Ur* urinary, *H* hair, *LOD* level of detection, *GSH* glutathione, *MDA* malondialdehyde, *SOD* superoxide dismutase, *TACs* total antioxidant capacity, *NF-kB* nuclear factor kappa-light-chain-enhancer of activated B cells, *Bcl2* B-cell lymphoma 2, *ALH* amplitude of lateral displacement of the head, *MAD* mean angular displacement, *LIN* linearity of the curvilinear trajectory, *STR* straightness of the average path, *WOB* wobble, *VCL* curvilinear velocity, *VSL* straight-line velocity, *PCB* polychlorinated biphenyls, *PFAS* perfluoroalkyl substances, *OP* organophosphates, *OC* organochlorines, *p,p′-DDT* p,p′-Dichlorodiphenyltrichloroethane, *p,p′-DDE* p,p′-dichlorodiphenyldichloroethylene, *HCB* hexachlorobenzene, *β-HCH* β-hexachlorocylohexane, *γ-HCH* γ-hexachlorocyclohexane, *VAP* average path velocity, *BCF* beat cross frequency

↑, positively correlated; ↓, negatively correlated; +, higher levels; −, lower levels

^*^Class 1: younger, higher sperm concentration and morphology; Class 2: older, lower sperm concentration and morphology

**Table 2:** Summary at a glance of clinical studies on the effects of air pollution on male fertility

Ref	Study design	N° of subjects Type of exposure	Age (years)	Biological matrix for dosage	Main findings
Cannarella R et al. (2019)	Retrospective cohort study	184 Environment Italy	M 37.6 ± 7.7	Sem	Total sperm count higher in patients living in low and middle-low industrial density areas
Bosco L et al. (2018)	Perspective study	**Group A:** 28 patients, exposed to air pollutants for both professional and residential reasons **Group B:**61 patients not exposed for residential reasons to air pollutants for work reasons; Groups A and B were patients enrolled in ART Clinic in Taranto, thus being mostly asthenozoospermic **Group C:** 70 patients exposed to air pollutants for residential reasons recruited at Gentile Laboratory (Pompei, province of Naples) **Group D:** 63 patients, 1 st control group with respect to groups A and B **Group E:** 105 patients, 2nd control group with respect to Group C Environment Italy	M 35.9 ± 5.7 (group A) M 37.06 ± 4.6 (group B) M 36.1 ± 4.2 (group C) M 38.5 ± 5.5 (group D) M 36.9 ± 5.6 (group E)	Sem	Sperm DNA fragmentation as a valuable early marker of pollution-related damage
Guan Q et al. (2020)	Retrospective cohort study	1955 Moderate to high exposure to air pollution China	M 28.9 ± 5.4	Sem	+ PM exposure throughout spermatogenesis during a long period reduce semen quality, sperm count and sperm concentration
Huang G et al. (2020)	Retrospective cohort study	1168 Sperm donors Environment China	M 26.0 ± 5.9	Sem	+ air pollutants exposures during sperm development may have an adverse effect on semen quality, especially for sperm count and motility
Lao XQ et al. (2018)	Cross-sectional study	6475 Participants Environment China	R 15–49	Sem	** + **PM2.5 air pollution is associated with a lower level of sperm normal morphology
Liu Y et al. (2017)	Retrospective cohort study	1759 Participants Environment China	M 34.4 ± 5.4 R 22.0–58.6	Sem	+ SO2 exposure adversely affects semen quality
Wu L et al. (2017)	Retrospective cohort study	1759 Environment China	M 34.4 ± 5.4 R 22.0–58.6	Sem	+ PM during sperm development adversely affects semen quality, in particular sperm concentration and count
Nassan N et al. (2018)	Retrospective cohort study	797 Environment Massachusetts		S Sem	Residential distance to major roadways was not related to semen quality or serum reproductive hormones

*PM* particulate matter, *SO* sulfur dioxide

R, range; M, mean; S, serum; Sem, semen; +, higher levels; −, lower levels

**Table 3:** Summary at a glance of clinical studies on the effects of global warming/environmental heat exposure on semen

Ref	Study design	Source/type of heat exposure	Type of patients N° of subjects/group	Age (years)	Main findings
Zhang X Z et al. (2013)	Retrospective study	Environmental	1866 sperm donors	m 24 (22–45)	Evident seasonal changes in semen parameter: The lowest level of semen volume, total sperm count, and normal morphology appeared in midsummer with AHT > 30 °C
Deng X et al. (2023)	Retrospective longitudinal study	Environmental (heat waves)	2183 sperm donation volunteers	M 26.2 ± 5.4	Exposure to heat waves was significantly associated with reduced semen quality
Veron G L et al. (2024)	Retrospective study	Environmental (heat waves)	54,907 men attending for fertility evaluation	M 41.26 ± 7.38	Semen from men exposed to heat waves has lower sperm number and abnormal morphology Heat wave length is negatively associated with semen quality Heat waves early exposure during spermatogenesis is detrimental for semen quality
Zhou T et al. (2020)	Prospective observational cohort study	Environmental	10,802 sperm donors	M 28.3	Inverted association between ambient air temperature and all semen parameters
Wang X et al. (2020)	Retrospective cross-sectional study	Environmental	1780 males	M 33.5 ± 5.1	An increase of 1 °C above the threshold detected accounted for a significant decrease of normal sperm morphology at different exposure windows before semen analysis
Veron G L et al. (2021)	Retrospective study	Environmental	11,657 males divided in seasonal groups: **Summer:** 2276 **Autumn:** 2947 **Winter:** 3456 **Spring:** 2978	-	Sperm quality parameters are negatively affected in summer when compared to winter Significant decrease in sperm kinematics between winter and spring
Zhang X et al. (2023)	Retrospective longitudinal cohort study	Environmental	11,050 sperm donors and volunteers	m 25.5	A 5 °C increase in ambient temperature exposure during the 0–90 days before ejaculation was significantly associated with reductions in semen quality parameters The associations were strongest with cumulative exposure over 90 days
Zhang X et al. (2024)	Prospective cohort study	Environmental	15,112 male partners of couples seeking fertility treatment	M 31	Heat waves had a broad and strong detrimental impact on all semen quality indicators, including both count and motility Exposure to very high temperatures caused significant declines in total motility, progressive motility, sperm concentration, and count

*M* mean, *m* median, *AHT* average highest temperature

## Endocrine disruptors and male fertility

### Heavy metals

Heavy metals are naturally occurring elements that have become widespread pollutants due to extensive anthropogenic emissions from agricultural, pharmaceutical, and industrial processes, one of the most common categories of pollutants spread in the environment. Heavy metals, particularly Pb, Cd, Cr, As, and Hg, have been classified as systemic toxicants and represent a substantial public health concern because of their persistence, bioaccumulation, and biomagnification through the food chain. Inhalation and ingestion of contaminated water and food are the primary exposure routes [3]. Although robust evidence supports the negative effect of heavy metals exposure on male reproduction, provided by experimental studies in animal models, epidemiological studies in humans remain limited and heterogeneous, both in study design and in the biological matrices used to assess exposure.

Ecological studies with no measurement of heavy metals in biological matrices highlighted that chronic low-level environmental exposure to heavy metals was associated with reduced sperm motility, particularly when comparing the 4th relative to the 1 st quartile of environmental exposure [4], whereas chronic high-level environmental exposure was associated with reduced sperm motility and concentration, abnormal sperm morphology, and reduced protamine/histone ratio [5]. A different ecological study specifically measuring Pb, Cd, and Cr in ambient fine particulate matter (PM2.5) found a positive association between Pb and Cd, but not Cr, with reduced sperm concentration [6].

The Longitudinal Investigation of Fertility and the Environment (LIFE) prospective study, performed on couples upon discontinuing contraception for purposes of becoming pregnant, found a significant association between male partner blood Pb, but not Cd or Hg, levels and a reduction in couple fecundity [7]. Conversely, environmental exposure to both Pb and Cd, when objectively determined by seminal concentration of metals, was associated with poor semen quality; in particular, higher seminal concentration of both heavy metals, but not of As and Hg, was associated with an increased prevalence of below-reference sperm viability in male partners of couples attending infertility clinics [8]. Interestingly, a different study demonstrated a significant association of semen Cd, but not Pb, levels with poor semen quality, independently from the agricultural or industrial residential address, suggesting that additional factors other than the environmental exposure to Cd (i.e., dietary antioxidants intake) might also contribute to such association [9].

Infertile men were found to display significantly higher blood and semen Cd levels [10, 11] and semen Pb levels [11]. In addition, higher blood and semen Cd levels and blood Pb levels were found in azoospermic and oligospermic infertile men, compared to normozoospermic infertile men [12, 13]. In infertile men, higher semen Pb and Cd concentration was associated with reduced sperm concentration, count and motility, and abnormal sperm morphology [11, 13], although results are more consistent for Cd than for Pb, across different studies. Moreover, higher semen Pb and Cd [14] and blood Cd [10] concentration was associated with oxidative stress and apoptosis markers.

Lastly, low-level environmental exposure to As, determined by urinary concentration of As species, was associated with idiopathic male infertility; in particular, higher urinary As levels were found in infertile men compared to fertile men [15].

The effects of Hg exposure on male fertility and specifically semen quality remain unclear, and results might be heavily affected by lack of control for potential confounders, such as fish consumption, known to exert a beneficial effect on semen parameters, as a major source of Hg and characterized by a high risk of cross-contamination with other pollutants [16]. The potential mechanisms underlying Pb and Cd reproductive impairment may include the following: impaired testosterone production driven by direct effects on Leydig cells and indirect effects on the hypothalamus-pituitary-testis axis; direct Leydig and Sertoli cell toxicity; structural and functional damage to the blood-testis barrier and testis vascular endothelium; zinc and selenium mimicry and displacement from antioxidant enzymes; and oxidative stress, DNA fragmentation, apoptosis, and inhibition of DNA damage repair systems within the testis [17, 18].

### Summary evidence

Clinical studies with different methodological settings consistently suggest that Pb and Cd exposure is associated with poor semen quality, and that a higher blood and seminal Pb and Cd burden is associated with male infertility, particularly in azoospermic and oligozoospermic infertile patients. Insufficient evidence on Cr, As, and Hg exposure prevents from drawing firm conclusions. Experimental studies in animal models suggest that potential mechanisms underlying Pb and Cd reproductive impairment may include the following: impaired testosterone production driven by direct effects on Leydig cells and indirect effects on the hypothalamus-pituitary-testis axis; direct Leydig and Sertoli cell toxicity; structural and functional damage to the blood-testis barrier and testis vascular endothelium; zinc and selenium mimicry and displacement from antioxidant enzymes; and oxidative stress, DNA fragmentation, apoptosis, and inhibition of DNA damage repair systems within the testis.

### Bisphenols

Bisphenols (BP), particularly bisphenol A (BPA), have been widely used as additives in the industrial production of plastic materials including polycarbonate-based and epoxy resin products such as food, beverages and microwave cooking containers, plastic dishes, and internal coatings in food cans; until 2011, they were also used in bottles. BPA is soluble in alcohol, ethers, and fats and may therefore contaminate food and drinks by direct contact, in particular after prolonged storage, exposure to high temperatures, and in the presence of foods with a high lipid component. Due to the massive use of plastics, in particular in the food industry, BPA is the chemical compound with the widest exposure in humans and poses a serious threat to public health. Ingestion of contaminated food and drinks, skin contact, and inhalation represent the main routes of exposure [19]. Robust evidence concerning the negative effect of BPA exposure on male reproduction has been provided by experimental studies in animal models, and quite consistent epidemiological data have been reported by human studies.

Environmental exposure to BPA, BPF, and BPS, determined by urinary concentration of BP, was not associated with seminal parameters in young men (18–20 years of age) from the general population in one observational study [20]; conversely, a different study on fertile men found a significant association between urinary BPA and decreased sperm concentration and impaired kinetic parameters, such as decreased sperm swing characteristics (ALH and MAD) and increased velocity ratios (LIN, STR and WOB), suggesting that BPA might affect spermatozoa functional competence to penetrate the zona pellucida [21]. Moreover, male partner urinary BPA level was associated with abnormal sperm morphology in a prospective, pre-conception cohort study [22].

BPA can be detected at comparable median levels in serum [23] and urine [24, 25] of both infertile and fertile men; nevertheless, high BPA concentrations were observed specifically in infertile men [23]. Urinary BPA level was negatively correlated with sperm concentration, motility, and normal morphology and positively correlated with DNA damage and seminal-plasma lipid peroxidation [25]. Higher urinary BPA levels were associated with increased risk of having below-reference sperm concentration, total count and motility [26], and total sperm sex chromosome disomy and spermatozoa immaturity, identified by a high DNA stainability [27], although other studies failed to find associations with seminal parameters [24, 27, 28]. Higher urinary BPS levels were associated with increased risk of having below-reference sperm motility [29]. A different study reported significantly reduced semen volume and sperm concentration among men with detectable urinary BPS concentrations, compared to men with non-detectable BPS; however, when stratifying men according to BMI, significantly reduced sperm concentration, total count, and motility were observed among men with detectable urinary BPS concentrations, compared to men with non-detectable BPS, only in overweight and obese men [30]. Moreover, semen BPA levels were negatively correlated with sperm concentration, total count [31], motility [25, 32], and normal morphology [25, 31, 32], while semen BPS levels correlated with semen quality, mainly volume and sperm total count [32].

Despite a consistent association of bisphenols exposure with poor semen quality, the LIFE prospective study failed to find an association between male partner urinary BPA levels and time to pregnancy [33], nor between semen BPA, BPF, and BPS levels and time to pregnancy [34]. Results on the lack of association between male partner urinary BPA levels and time to pregnancy were confirmed by the Home Observation of Periconceptional Exposures (HOPE) study on healthy pregnancy planners [35]. Interestingly, the LIFE study reported no correlation between urinary BPA levels and seminal parameters and a negative correlation between urinary BPA levels and spermatozoa DNA fragmentation, though these results may be biased by low urinary BPA concentrations and the use of next-day semen analysis [36].

### Summary evidence

Clinical studies consistently suggest that bisphenols exposure, particularly BPA, is associated with poor semen quality, reduced antioxidant levels, and increased sperm sex chromosome disomy, spermatozoa immaturity, DNA damage, and seminal-plasma lipid peroxidation, although few studies did not confirm these associations; moreover, high urinary BPA levels are associated with male infertility. Nevertheless, the quite consistent effect on seminal parameters is not reflected by a clear association with fertility outcomes. Experimental studies in animal models suggest that potential mechanisms underlying BPA reproductive impairment may include the following: impaired testosterone production driven by direct effects of BPA on Leydig cells and indirect effects on the hypothalamus-pituitary-testis axis; direct Leydig and Sertoli cell toxicity; major impairment of the spermatogenetic process and germ cell maturation; reduced acrosomal integrity; altered germ cell glucose homeostasis; and oxidative stress, DNA fragmentation, and apoptosis within the testis.

### Phthalates

Phthalates, or phthalate esters, are a wide class of anthropogenic plasticizers, among which di(2-ethylhexyl) phthalate (DEHP) is the most commonly used. DEHP is routinely employed in the production of polyvinyl chloride (PVC), the world’s second most-used plastic. Phthalates are widely used in food packaging, blood storage bags, intravenous tubing, and pesticides and, until 2008, in children’s toys. Like BP, phthalates can contaminate food via direct contact and are known to bioaccumulate in several fish species. Ingestion of contaminated food represents the main route of exposure, although for some phthalates, the greatest exposure occurs through personal care products [37]. Robust evidence concerning the negative effect of phthalates exposure on male reproduction has been provided by experimental studies in animal models, demonstrating that these compounds often act in complex mixtures, exerting synergistic effects on common molecular targets. Human studies have quite consistently supported an overall detrimental effect of phthalates on male fertility.

Ecological studies without direct measurement of phthalates or their metabolites in biological samples have shown that chronic low-level environmental exposure to phthalates was associated with reduced semen volume and sperm motility and increased prevalence of azoospermia [4]. One prospective study attempting to test the hypothesis that specific environmental factors might explain downward trends in semen quality over time demonstrated that downward trends in both sperm concentration and morphology over time were attenuated by 19% when including urinary phthalate levels. In particular, urinary mono(2-ethyl-5-carboxypentyl) phthalate (MECPP) accounted for most of the contribution towards decreasing sperm concentration and urinary monoisobutyl phthalate (MIBP) and mono(3-carboxypropyl) phthalate (MCPP) levels towards decreasing sperm normal morphology [38]. Different cross-sectional studies corroborate an involvement of phthalates exposure in poor semen quality. In the general population, urinary DEHP metabolite levels—particularly MECPP, mono(2-ethyl-5-hydroxyhexyl) phthalate (MEHHP) and mono(2-ethyl-5-oxohexyl) phthalate (MEOHP)—were negatively correlated with sperm motility; moreover, urinary DEHP metabolite levels—particularly mono(2-ethylhexyl) phthalate (MEHP)—were positively associated with spermatozoa immaturity, as measured by DNA stainability [39]. In different studies on males from infertile couples, including both a male or a female factor of infertility, urinary phthalate metabolite levels were associated with reduced sperm concentration (monobenzyl phthalate (MBzP), MEHP) [40, 41], motility (MBzP, MEHP) [39], morphology (monobutyl phthalate (MBP), (MIBP)) [41], INSL3 (MBzP, MEHP) [40, 41], acrosin activity (MBP, MIBP, MEHP) [41], and increased spermatozoa DNA fragmentation 40, and seminal phthalate metabolite levels were associated with reduced sperm concentration (MEHP, MEHHP), motility (monoethyl phthalate (MEP), MEHHP, MECPP), normal sperm morphology (MEP), and INSL3 (monomethyl phthalate (MMP), MEP) [40]. In a study performed on couples upon discontinuing contraception for purposes of becoming pregnant, male partners’ urinary levels of phthalates were associated with reduced sperm count and concentration (mono[2-(carboxymethyl)hexyl] phthalate (MCMHP), MEHHP, MBzP, monononyl phthalate (MNP)) and motility (MCMHP, MEOHP, MECPP, MCPP), and increased abnormal sperm morphology (MCMHP, MNP) [42].

Infertile men were found to display significantly higher semen dioctyl phthalate (DOP) [43, 44], diethyl phthalate (DEP), benzyl butyl phthalate (BBP), dibutyl phthalate (DBP) and DEHP levels [44], and higher blood DEHP, DBP, diisobutyl phthalate (DIBP), Bis(2-ethylhexyl) isophthalate (BEHIP), butyl phthalyl butyl glycollate (BPBG), dipentyl phthalate (DPP), diisooctyl phthalate (DIOP), diisoheptyl phthalate (DIHP), dimethyl phthalate (DMP), diisononyl phthalate (DINP), butyl isooctyl phthalate (BIOP), dimethoxy octyl phthalate (DMOP), and dicyclohexyl phthalate (DCHP) levels, and urinary MBP and MEHP levels [45] than those observed in fertile men, although no correlation was found with any seminal parameter [43, 45]. Conversely, a different study highlighted that higher urinary levels of the phthalate metabolites were associated with below-reference sperm concentration and total count (MBP) and normal morphology (MEHP) [46]; moreover, mixtures of phthalate metabolites in seminal plasma were negatively associated with the expression of spermatogenesis-related miRNA106a, with MEHP likely representing the major contributor to such effect [47]. Consistently, higher semen levels of phthalate metabolites were found to be associated with reduced semen volume (MBP, MEHP, MEHHP, MEOHP) sperm kinetic parameters (MBzP, MEHP) and increased abnormal sperm morphology (MBzP) in male partners of sub-fertile couples [48]. Similar evidence was reported in fertile men, displaying no association of urinary DEHP, DBP, DEP, and di-n-octyl phthalate (DOP) levels with any seminal parameter and a negative correlation of urinary MBzP with sperm motility [49].

Lastly, studies on the Fudan Preconceptional Offspring Trajectory Study (PLOTS) [50] and LIFE [7, 33] prospective cohorts found a significant association between male partner urinary total phthalates and MEHP and MCMHP [50] levels and MBzP, MMP [7, 33] levels, and a reduction of couple fecundity and longer time to pregnancy, whereas contrasting data for male partners blood MEHP levels [51] and no association for semen level of MECPP, MCMHP, MEOHP, MEHHP, MCPP, MMP, MEP, MBP, MIBP, mono-hexyl phthalate (MHxP), mono-cyclohexyl phthalate (MCHP), mono-octyl phthalate (MOP), mono-isononyl phthalate (MINP), MBzP, mono-(8-methyl-1-nonyl) phthalate (MIDP), and phthalate diesters DMP, DEP, DIPB, DBP, BBP, Di-n-hexyl phthalate (DNHP), dicyclohexyl phthalate (DCHP), DEHP, and DOP [34] were reported.

Few reports suggested a positive association of low-level environmental phthalate exposure, objectively determined in biological matrices, and sperm motility [52] and would require further investigation in order to dissipate such inconsistencies. Furthermore, recent evidence suggests that evaluating urinary phthalate metabolites in their conjugated and free form, rather than total levels, may provide more accurate biomarkers of exposure and health effects [52]; unfortunately, almost the totality of studies so far disregarded this concept.

### Summary evidence

Clinical studies consistently suggest that phthalates exposure is overall associated with poor semen quality and male infertility, although no firm association with fertility outcomes can be drawn. Nevertheless, it must be stressed that the interpretation of data is extremely complicated by heterogeneous study designs and the enormous number of different phthalates and phthalates metabolites that would need to be simultaneously addressed. Moreover, available data denote complex interactions, and differential associations of the different phthalates or phthalates metabolites have been highlighted, strictly depending on the specific endpoint, and strongly suggest that the choice of biological matrices and species to be determined needs to be carefully addressed. Experimental studies in animal models suggest that potential mechanisms underlying phthalates’ reproductive impairment may include impaired testicular steroidogenesis, morpho-structural alterations of the seminiferous tubules, disrupted spermatogenesis up to complete absence of germ cells, Sertoli cells adherent junctions damage and reduced Sertoli cells viability, and oxidative stress.

#### Polychlorinated biphenyls

Polychlorinated biphenyls (PCBs) are a stable group of chemicals composed of chlorine atoms attached to biphenyl rings. Their heat resistance and electrical insulating properties allow for a wide use in industrial applications until their environmental persistence and toxic effects were recognized. Although PCBs have been banned in many countries, their long half-life and persistence in the environment result in bioaccumulation in animal and human fatty tissues [53, 54]. PCBs could affect androgen activity, disrupting testosterone production and spermatogenesis. In fact, Den Hond et al. [28] concluded in their study that exposure to PCBs in 163 subfertile men was associated with altered fertility outcomes. Further evidence of PCB-induced endocrine disruption comes from Petersen et al. [55], who evaluated 266 fertile men with elevated PCB exposure. Their study found that high PCB concentrations were associated with increased serum concentrations of SHBG and FSH, without impairing semen quality. Tøttenborg et al. [56] evaluated prenatal exposure to PCBs and male reproductive health, reporting that higher maternal exposure to PCB was linked to a lower total sperm count, increased FSH values, and risk of small testicles, together with a lower sperm fragmentation index. Cai et al. [57] investigated the relationship between PCB exposure and spermatozoa prosaposin levels, a protein involved in sperm maturation. They found that reduced prosaposin level in spermatozoa was significantly associated with PCB exposure, suggesting that PCBs may interfere with sperm maturation processes. A similar conclusion was reached by Moltó et al. [58], examining PCB levels in the serum of male subjects. Their results showed a correlation between elevated PCB concentrations and reduced sperm quality, particularly in terms of sperm motility and morphology. This study reinforces the hypothesis that exposure to PCBs may have detrimental effects on male reproductive health. The same group carried out a case–control study including two groups of patients [59]. Total concentrations of PCBs were significantly higher in the low semen quality group than in the normal semen quality group. Finally, a case-cohort study conducted by Desalegn et al. [60] found that prenatal exposure to PCBs may increase the risk of cryptorchidism as well as reproductive disorders in male offspring, with long-term implications for male fertility.

### Summary evidence

Studies on PCB exposure to male impairment of fertility support that PCBs play a fundamental role as EDC in the environment. Exposures to PCBs relate to altered quality of sperm, disrupted hormonal homeostasis, reduced fertility capacity, and risk for cryptorchidism. Underlying mechanisms responsible for these impairments appear to be disruption in testosterone synthesis, spermatogenesis, and spermiogenesis. Furthermore, studies have shown that the impact of PCB exposure may be transgenerational, where not only the individuals exposed but also their progeny are affected.

### Perfluoroalkyl substances

Perfluoroalkyl substances (PFAS) are a group of synthetic chemicals used in various industrial applications and consumer products [61, 62]. High concentrations of perfluorooctane sulfonate (PFOS) have been detected in the blood of retired fluorochemical industry workers [63]. These chemicals are persistent in the environment and human tissues and are therefore also called “forever chemicals” [64]. Due to their widespread use and persistence, PFAS are commonly found in water, air, soil, food, and in the blood, where they also accumulate in various organs; it has been reported that they cross the blood–brain barrier, placenta, and blood-testis barrier. Their potential effects on reproductive health have increased in recent years. Perfluorooctanoic acid (PFOA) and PFOS, the most widely studied PFAS compounds, have been shown to affect hormone release, including testosterone. In this regard, [65] conducted a study on men from different regions of Italy, finding that exposure to PFAS was higher in men from the metropolitan area and was associated with altered gene expression in nuclear receptors involved in fertility, including androgen receptors. Similarly, Di Nisio et al. [66] demonstrated PFOA interference on the androgen receptor with consequent reduction of sperm number and quality and a raise in LH. Further evidence was reported by Steves et al. [67], demonstrating that PFAS exposure impaired human spermatogenesis using a stem-cell-derived model; PFAS may directly impact spermatogonia stem cells, disrupting the early stages of sperm development with a long-term impact on male fertility.

Several studies have found that PFOA and other PFAS chemical exposure is linked to reduced sperm quality, including motility, concentration, and morphology. For instance, Alamo et al. [68] assessed the in vitro effects of PFOA on human sperm function and found significant alterations in total and progressive sperm motility, impaired chromatin compactness, and increased sperm lipid peroxidation and mitochondrial superoxide anion levels, suggesting that even low-level environmental exposure to PFOA could compromise male fertility. Šabović et al. [69] reported that reduced sperm motility is caused by PFA interference on sperm membrane, leading to impaired chromatin compactness and increased sperm lipid peroxidation and mitochondrial superoxide anion levels. Furthermore, Forthun et al. [70] demonstrated that the effects of PFAS exposure in Norwegian boys and male youths were quantified. Results of the research indicated higher PFAS levels were associated with interrupted pubertal timing, with a possible effect on reproductive function in adulthood. The current research highlights the necessity of determining the long-term effects of PFAS exposure in vulnerable developmental stages on male fertility. In addition, the same team [71] investigated the relation between PFAS exposure and onset of puberty among Norwegian boys, and they determined that increased levels of PFAS were correlated with delayed onset of puberty. Given that puberty is a key developmental window for establishing male reproductive capacity, such delays may result in persistent impairments in sperm production and quality.

### Summary evidence

A correlation of PFAS exposure with male fertility impairment appears to be consistent, inducing reduced sperm quality. As PFAS continue to persist in the environment as well as in human tissues, it becomes very important to understand the entire scenario of their effect on reproductive health. Future studies should focus on longitudinal evaluations to elucidate the long-term reproductive effects of PFAS, as well as on studies of the mechanisms of PFAS-induced reproductive toxicity, mainly in vulnerable populations such as children and adolescents.

### Dioxin

Dioxins, primarily formed as industrial process by-products such as waste incineration and production of certain chemicals, are persistent organic pollutants accumulated in the environment and in human body tissues [72]. As endocrine disruptors, they interfere with hormonal regulation relevant to reproductive health. The effects of dioxins on male fertility, particularly in the presence of subfertility and infertility, have been a subject of growing scientific investigation over the last few years. In fact, new evidence shows that exposure to dioxins can negatively influence sperm quality, testicular function, and fertility outcomes in men. Den Hond et al. [28] indicate that male subfertility patients exposed to dioxins showed abnormal hormonal profiles and lower fertility potential. This case-control study supports the role of dioxins as contributing factors to male subfertility and underscores the need for ongoing research on the environmental impact on reproductive function. Eskenazi et al. [73] conducted a study in Seveso, Italy—an area heavily contaminated by dioxin following a 1976 industrial accident—and analyzed data from 677 offspring (341 females and 336 males). Their findings revealed that maternal dioxin exposure was associated with lower fecundability and higher rates of infertility in both mothers and their daughters. These results provide compelling evidence of the long-term, intergenerational effects of dioxin exposure on reproductive health, even more in the female population. Again, Paul et al. [59] explored the relationship between serum concentrations of dioxin-like PCB and sperm maturation in men attending fertility clinics. The study found that elevated levels of these compounds were associated with delayed post-testicular sperm maturation, suggesting that exposure to dioxins and similar chemicals can compromise male reproductive function. Similarly, Moltó et al. [58] investigated the levels of dioxin-like PCBs in the serum of male fertility patients. They found a significant correlation between higher PCB levels and lower sperm quality, further implicating environmental pollutants in male fertility issues. Collectively, these findings reinforce the conclusion that dioxins may exert adverse effects on key sperm parameters such as motility, morphology, and concentration.

### Summary evidence

Literature evidence presents a high correlation between dioxin exposure and male fertility compromise, decline in sperm quality, and reduced fertility outcomes. Even though the specific mechanisms remain to be studied, it is evident that environmental exposure to dioxin is a critical risk factor for male reproductive health. Dioxin exposure effects in the long term, possible intergenerational effects, and effective interventions to prevent reproductive health compromise are subjects to be studied in the future.

### Pesticides

Pesticides (PSTs) is an umbrella term comprising a very wide and heterogeneous group of chemical substances used as herbicides, insecticides, nematicides, fungicides, molluscicides, rodenticides, bactericides, virucides, disinfectants, and sanitizers; major classes of pesticides are represented by organophosphates (OP) and organochlorines (OC). OPs are a class of organophosphorus compounds, for the vast majority used as insecticides; leakage into the environment through volatilization and abrasion may easily occur and, although OPs are rapidly degraded, small amounts may enter the food chain. Therefore, ingestion of contaminated food and water, skin contact, and inhalation represents the main routes of exposure. Since the 1990 s, increasing restrictions on OP use for crop protection have been introduced, and use of OP has decreased considerably since then [74]. Differently from OP and OC, organic compounds that contain one or more carbon–chlorine bonds are persistent organic pollutants used in the production of PVC, as solvents for degreasing and dry cleaning, and as insecticides for the control of different insect-borne human diseases, as well as for insect control in crops; OC may easily enter the food chain and bioaccumulate in body fat for many years. Ingestion of contaminated food and water and inhalation represent the main routes of exposure. Despite being among the most widely used OC, due to their high efficacy and low cost, dichlorodiphenyltrichloroethane (DDT) and its congener p,p′-dichlorodiphenyldichloroethylene (p,p′-DDE) were banned in the 1970s–1980s; nevertheless, concerns for human health may persist due to potential long-term OC adverse effects [75]. Although the majority of available human studies have been performed in occupationally exposed workers, epidemiological evidence quite consistently supports an overall detrimental effect of environmental exposure to pesticides on male fertility also in the general population, in terms of impaired semen quality and spermatozoa DNA fragmentation.

A cross-sectional study focusing on exposure to pesticides from fruit and vegetable intake highlighted that in men consuming crops classified in the high pesticide residue fruits and vegetables group, according to data from the United States Department of Agriculture (USDA) Pesticide Data Program (PDP), pesticide exposure was associated with decreased sperm total count, motility, and normal morphology [76].

Chronic exposure to OP and OC was found to be associated with decreased semen quality. A comparative study performed in rural farmers and urban men found that exposure to OP, determined by serum butyrylcholinesterase (BchE) as a proxy index of exposure, was associated with decreased sperm concentration and motility and decreased seminal antioxidant activity in farmers, compared to the urban region population [77]. Moreover, in a case-control study performed in male partners of infertile couples and normozoospermic men, OP exposure, determined by serum pseudocholinesterase and acetylcholinesterase as a proxy index of exposure and urinary levels of OP metabolites dialkyl phosphate (DAP), was significantly more prevalent in cases than in normozoospermic men and was negatively correlated with sperm concentration, motility, and morphology [78]. Lastly, a negative association was observed between urinary concentration of non-persistent OP metabolites and sperm motility, normal morphology, and spermatozoa DNA damage [79].

A study performed on the male general population found that exposure to OC, determined by serum p,p′-DDE levels, was associated with decreased sperm motility and kinetic parameters [80]. Moreover, a longitudinal study performed on boys from the general population reported that higher peripubertal serum levels of hexachlorobenzene (HCB) and β-hexachlorocyclohexane (β-HCH) were associated with decreased semen volume, and higher serum levels of p,p′–DDE with decreased sperm motility later in life, highlighting the detrimental long-term effects of exposure to OC on semen quality [81]. Interestingly, the LIFE study reported positive associations between serum levels of β-HCH, p,p′-DDT, o,p′-DDT, and p,p′-DDE and sperm motility and kinetic parameters, though these results may be biased by low serum concentrations of OC and the use of next-day semen analysis [82].

### Summary evidence

The environmental exposure to pesticides, both occupationally and not, is associated with impaired male reproductive health. Several studies have reported a reduction of sperm concentration, motility, an increase of abnormal sperm morphology, and sperm DNA fragmentation. Finally, early-life exposure has been related to long-term effects on semen parameters in adulthood.

## Air pollution and male fertility

Air pollutants have been associated with impaired semen parameters. In this regard, two Italian studies found respectively a total sperm count higher in patients living in low and middle-low industrial density areas compared with that of men living in middle and high ones [83] and identified sperm DNA fragmentation as a valuable early marker of pollution-related damage [84]. Five studies were conducted in China [85–89], suggesting that ambient particulate matter (PM) exposure during sperm development adversely affects semen quality, including concentration [85, 89], motility [86, 88], and morphology [87]. Conversely, Nassan et al. [90] evaluated the associations of residential distance to major roadways in Massachusetts with semen quality and found no significant relationship. Interestingly, a recent meta-analysis conducted on 17 articles, with a total of 24,065 participants, with a range of age from 18 to 40 years old, evaluated the effect of air pollution on sperm parameters [91]. Despite the studies heterogeneity, the authors found that a higher exposure to outdoor air pollution was associated with a significant reduction of semen volume (WMD, −0.13 mL; 95% CI −0.21 to −0.05; *P* = 0.001; *I*^2^ = 32.1%), sperm concentration (WMD, −12.41 × 106/mL; 95% CI −23.29 to −1.53; *P* = 0.03; *I*^2^ = 98.7%), both total (WMD, −5.96%; 95% CI −10.76 to −1.16; *P* = 0.01; *I*^2^ = 96.2%) and progressive motility (WMD, −4.89%; 95% CI −9.23 to −0.55; *P* = 0.03; *I*^2^ = 98.0%), and normal morphology rate (WMD, −2.64%; 95% CI −4.36 to −0.92; *P* = 0.003; *I*^2^ = 94.6%). Furthermore, they found a significant increase in DNA fragmentation index (WMD, 5.41%; 95% CI 3.24 to 7.59; *P* < 0.001; *I*^2^ = 70.4%).

## Summary evidence

Air pollution negatively impacts semen quality through impairment of all semen parameters.

## Impact of temperature global warming on male fertility

Spermatogenesis is a complex and finely regulated process that could be affected by several external and environmental factors. It has long been known that spermatogenesis is a temperature-dependent process since testicular function needs an ideal temperature of 2–3° below body temperature. For example, spermatogenesis is defective in undescended testis, and a possible explanation could be the exposition to the higher intrabdominal temperature [92]. Occupational studies have identified an increased risk of impaired semen quality among workers exposed to heat, such as firefighters [93] and steel industry workers [94]. Nonetheless, the confounding effects of co-exposure to toxic substances in these settings cannot be excluded. Additionally, lifestyle factors may contribute to testicular heat stress, including frequent sauna use, hot baths, prolonged sitting with laptops on laps, use of seat heaters, or tight-fitting underwear [95, 96]. Experimental studies on healthy volunteers exposed to artificially induced scrotal heat stress have supported these findings [94, 95]. In one such study, Zhang et al. enrolled 19 healthy men who wore an electrically heated scrotal heat stress (SHS) bag (40–43 °C) for 40 min, twice a week, over 3 months. The authors observed a significant decline in sperm concentration, motility, and normal morphology after just 1 month of SHS exposure, along with an increase in white blood cells in the semen. After 3 months, further reductions in semen volume were found, together with increased sperm DNA fragmentation, loss of membrane integrity, and elevated caspase-3 activity [97]. In a follow-up study in 2018, the same group found significant alterations in hormonal parameters, including LH, FSH, and testosterone, after 3 months of SHS exposure using a warming belt [98].

Experimental studies showed that exposure to heat waves is strongly related to a decline in semen quality, including reduced sperm count, motility, and morphology [99]. A heat wave is a climatic phenomenon characterized by prolonged periods of unusually high temperatures, typically defined relative to a specific temperature threshold and duration. Since heat waves are most frequent in the summer, these findings align with previous research that highlights seasonal variations in semen quality [100]. In 2013, Zhang et al. reported that semen volume, sperm concentration, motility, and percentage of spermatozoa with normal morphology are inversely related to fluctuations in peak temperatures, with significantly lower parameters observed in midsummer [101]. Similarly, in Wuhan, China, Zou et al. investigated the effects of ambient temperature on semen quality in 10,802 sperm donation volunteers. They reported an inverted U-shaped exposure–response relationship between temperature and both sperm count and motility, identifying an optimal exposure threshold of 13 °C (55.4 °F) during the 0–90 days preceding semen collection. These findings suggest that both low and high temperatures contribute to a decline in semen quality [102].

Another study from China, Zhang et al. [103] demonstrated a negative relationship with a linear correlation between ambient temperature and semen quality, concluding that ambient temperature may be beyond a possible level of cold exposure that affects semen quality. A study with 15,112 subjects and 28,267 semen samples emphasized that exposure to high temperatures negatively affects sperm quality. At the 98th percentile, all six sperm parameters were affected, and sperm count was particularly susceptible after exposure for 3 days. At even higher temperatures (99th percentile), negative effects were more potent, with more severe impairment in progressive motility and sperm count [104]. Another study of 1780 men from Wuhan, China, also provided further evidence of an ambient temperature threshold effect on sperm quality. An increase of 1 °C above the threshold detected accounted for a significant decrease of normal sperm morphology at different exposure windows before semen analysis, with the most pronounced effects at the more proximally exposed times [105]. In 2021, Veron et al. conducted a study evaluating the impact of various meteorological variables on semen quality in men from Argentina. Of the 11,000 samples, they found a decline of semen parameters in summer compared to winter, with significant decreases also observed between winter and spring. Their analysis showed that changes in sunshine duration and humidity notably influenced sperm concentration, motility, and vitality [106]. In 2024, the same group published a study analyzing 54,926 men in Buenos Aires, Argentina, from 2005 to 2023 and found that exposure to heat waves (defined as ≥ 3 consecutive days with temperatures of 32.3 °C or higher and 22 °C or higher) during spermatogenesis negatively impacted semen quality. Men exposed to heat waves had lower sperm concentration, count, and normal morphology, with the strongest effects observed in years with frequent heat waves. Longer heat wave exposure was more detrimental, particularly during early spermatogenesis (64–90 days before semen collection) [100].

In this section, we also considered studies not directly focused on global warming to illustrate the impact of elevated temperatures on male fertility. Investigating the specific effects of global warming on fertility remains challenging due to several methodological limitations in the existing literature. Moreover, individual-level data on indoor environments (such as air conditioning or workplace heat exposure) were generally not available. Nonetheless, these studies also demonstrate notable strengths. Among them are the use of longitudinal designs and large cohorts with extended follow-up periods, the analysis of multiple exposure windows to encompass the full duration of spermatogenesis, and the application of sophisticated statistical techniques which enhance the robustness of the findings.

## Summary evidence

Heat exposure adversely affects sperm quality and male fertility, although the mechanisms are not yet clear. Male infertility can be a significant consequence of global warming, and thus, greater attention should be given to this growing issue.

The studies we have presented exhibit several limitations, including selection bias and, sometimes, the absence of repeated semen quality assessments. Additional limitations are the absence of consideration for potential co-exposures other than heat, the inclusion of heterogeneous populations, and the estimation of heat exposure based solely on ecological weather data. Finally, additional research is required to determine the global impact of climate change on male fertility.

## Transgenerational effects

The exposure to the environment, mainly EDCs, may induce epigenetic modifications, which can directly modulate the regulation of genetic processes, DNA methylation alterations, and histone modifications that are heritable across generations, raising concerns about long-term reproductive consequences [107]. Studies conducted on animals have demonstrated that prenatal or early-life exposure to substances such as dioxins can lead to transgenerational effects on male fertility, including altered sperm parameters and epigenetic reprogramming of the germline [108]. Although data on humans remain limited, the potential for irreversible reproductive harm underscores the need for precautionary regulatory policies and comprehensive monitoring of EDC exposure. Incorporating the transgenerational paradigm into risk assessment frameworks could significantly strengthen public health protection strategies.

## Limitations and future perspectives

Despite the growing body of literature, several methodological limitations persist. Firstly, the majority of the studies are observational in nature (i.e., ecological studies), thus limiting causal inference and confounding. In this regard, common confounders, such as dietary habits, smoking, occupational exposures, and socio-economic status, are often not assessed. Secondly, substantial heterogeneity exists in exposure assessment methods, particularly for chemicals such as BPA, phthalates, and PFAS, due to the variety of sampling matrices and timing, complicating comparisons across studies.

Additionally, sample sizes in several studies are limited, which reduces statistical power. Several investigations also rely on self-reported outcomes or semen parameters assessed in subfertile populations, which may not be generalizable to the broader male population. Moreover, publication bias toward studies reporting positive associations cannot be excluded.

Another critical point is the presence of non-monotonic dose–response relationships for many EDCs. This non-linear behavior complicates toxicological interpretation and identification of safe exposure thresholds.

From a review perspective, the absence of a formal risk of bias assessment represents a further limitation.

Future research should prioritize prospective designs with careful attention to methodology, using standardized exposure assessments and sufficient power, and should aim to elucidate mechanistic pathways through which environmental factors influence male reproductive health. Clinicians should consider the geographic origin of study participants—especially those from areas known to be environmentally at risk—as well as conduct a comprehensive assessment of occupational exposures, lifestyle factors, and confounding factors. Studies assessing the impact of environmental pollution on the reproductive health of the population, with a comparison of areas with a high rate of detected pollution versus areas with normal or low rates, should be encouraged.

## Conclusions

**Fig. 1:**
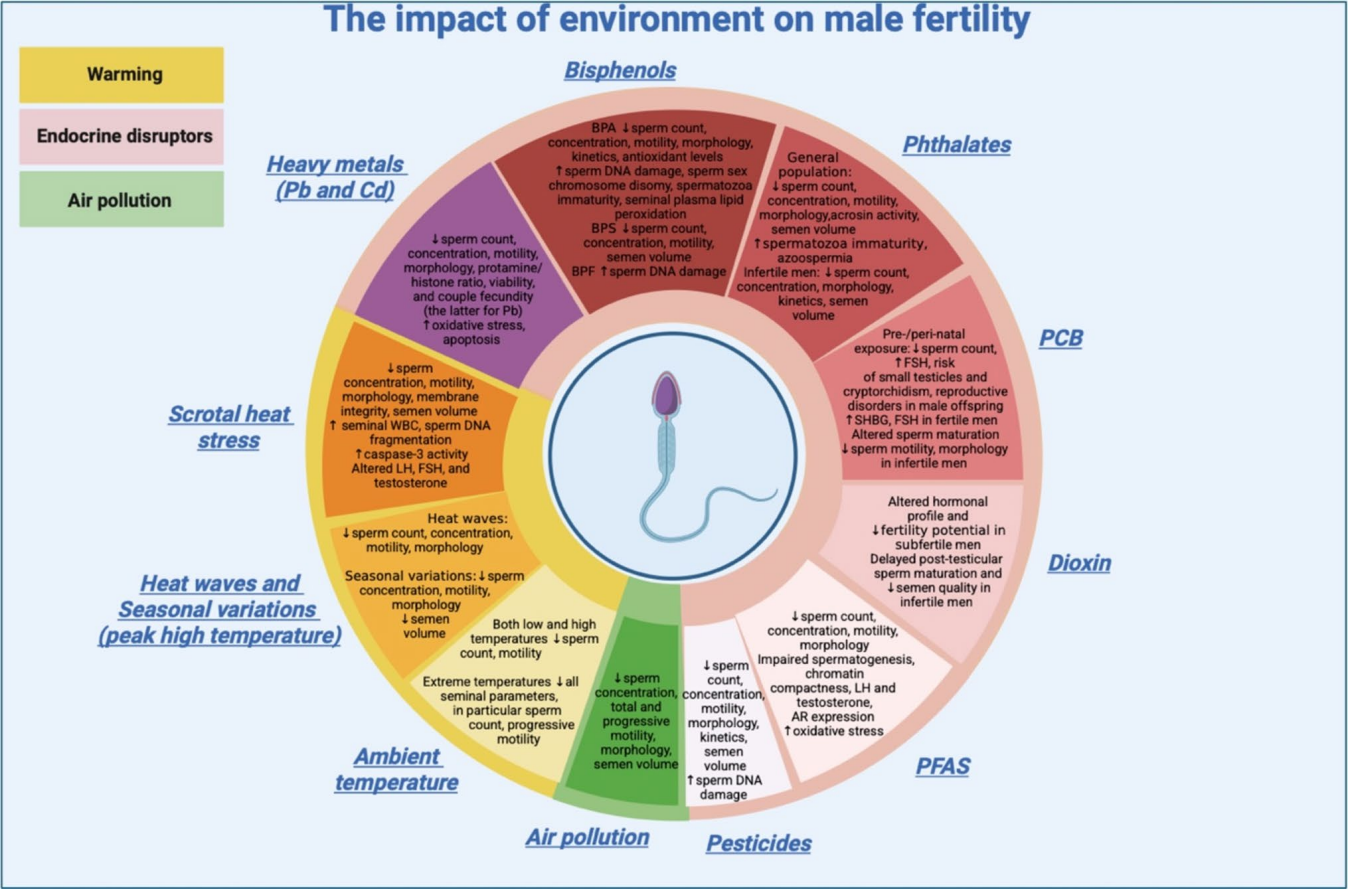
Summary of the potential effects of warming, endocrine disruptors, and air pollution on male fertility. PCB, polychlorinated biphenyls; PFAS, perfluorinated alkylated substances; AR, androgen receptor; WBC, white blood cell; BPS, bisphenol S; BPF, bisphenol F

The effects of environment on male fertility could be summarized into three major groups of stressors: endocrine disruptors, air pollution, and global warming (Fig. 1).

Based on the current body of evidence in the literature, the following conclusions can be drawn:

- Exposure to endocrine-disrupting chemicals, environmental pollutants, and heat contributes to a decline in reproductive health.
- Research should focus on the role of environmental factors in male hypogonadism, impaired spermatogenesis, genital abnormalities, and transgenerational effects.
- Multidisciplinary clinical research integrating expertise from andrology, gynecology, embryology, and social sciences is essential to investigate fertility trends and their underlying causes. Collaborative research including experts in biochemistry and statistics is highly recommended, due to the constant simultaneous co-exposure to cocktails of compounds potentially exerting additive/synergistic or antagonistic effects and to the frequently observed complex non-monotonic dose-response relationships.
- Public education efforts should be strengthened to raise awareness about the potential risks posed by environmental factors on reproductive health; in the same way, efforts to reduce contamination in the environment and limit exposure to chemicals are essential to protect reproductive health, particularly in vulnerable populations such as children and adolescents.
- Interdisciplinary collaboration among health care professionals and experts in the field of environmental pollution, as well as local environmental governance stakeholders, is both essential and non-negotiable.

## Data Availability

Not applicable.
